# General Intelligence

**Published:** 1844-09

**Authors:** 


### GENERAL INTELLIGENCE.

*Medical Schools.*—The Jefferson Medical College, Philadelphia, announces its Winter Course under the same Faculty in whose hands its popularity has so greatly increased in the last year. A change for the better it would be difficult to make.

The College of Physicians and Surgeons, New York City, with Dr. Alexander H. Stevens, President, makes its announcement under favorable auspices. The class of last year is represented as one-half larger than that of the year previous.

In the University of New York Medical Department there has been no changes in the Faculty of last year. A large appropriation has been made to this Institution by the Legislature of the State.

In the Faculty of Transylvania University, several changes have occurred. Dr. James M. Bush, formerly adjunct to Dr. Dudley, has been entrusted with the chair of Special and Surgical Anatomy. Dr. Dudley retains the chair of Surgery alone. Lotan G. Watson, M. D., has been appointed to the chair of Theory and Practice, and Leonidas M. Lawson, M. D., editor of the Western Lancet, to the chair of General and Pathological Anatomy and Physiology. In other departments there has been no change.

In the Medical Department of the St. Louis University, there has been a new organization. The Faculty is as follows:

"Charles A. Pope, M. D., Professor of Special, General and Surgical Anatomy; J. V. Prather, M. D., Professor of the Principles and Practice of Surgery, and Dean of the Faculty; J. W. Hall, M. D., Professor of Physiology, Pathology, and Clinical Practice; M. L. Linton, M. D., Professor of the Principles and Practice of Medicine; J. G. Norwood, M. D., Professor of Materia Medica, Therapeutics, and Medical Jurisprudence; A. Litton, M. D., Professor of Chemistry and Pharmacy; M. M. Pallen, M. D., Professor of Obstetrics, and the Diseases of Women and Children; W. D. Stirman, M. D., Prosector."

The Willoughby University, upon Lake Erie, announce some new appointments. As at present organized, their Faculty embraces the following names:

"Amasa Trowbride, M. D., Professor of Surgery; Geo. McCook, M. D., Adjunct Professor of Surgery; Henry H. Childs, M. D., Professor of Obstetrics, and Diseases of Women and Children; James Quackenboss, M. D., Professor of General and Special Anatomy and Physiology; Robert H. Paddock, M. D., Professor of Chemistry, Pharmacy and Materia Medica; John Butterfield, M. D., Professor of Theory and Practice, and of General and Special Pathology; Isaac J. Allen, M. D. and Counselor at Law, Professor of Medical Jurisprudence."

---

*Notice to Readers and Correspondents.*—In addition to the exchanges, the receipt of which we have already acknowledged, we have received The American Journal of Insanity, Utica, N. Y.; The Western Journal of Medicine and Surgery, Louisville, Ky.; The New York Journal of Medicine and the Collateral Sciences.

We have also received the Annual Announcement of the Jefferson Medical College, Philadelphia; University of New York, Medical Department; College of Physicians and Surgeons, New York City; Transylvania University, Lexington, Ky.; St. Louis University, Mo.; and the Willoughby University, Ohio.

